# The CII-specific autoimmune T-cell response develops in the presence of FTY720 but is regulated by enhanced Treg cells that inhibit the development of autoimmune arthritis

**DOI:** 10.1186/s13075-015-0909-6

**Published:** 2016-01-12

**Authors:** David C. Miller, Karen B. Whittington, David D. Brand, Karen A. Hasty, Edward F. Rosloniec

**Affiliations:** Department of Medicine, University of Tennessee Health Science Center, Memphis, TN 38163 USA; Department of Molecular Sciences, University of Tennessee Health Science Center, Memphis, TN 38163 USA; Memphis VA Medical Center, 1030 Jefferson Avenue, Memphis, TN 38104 USA; Department of Orthopaedic Surgery, University of Tennessee Health Science Center, Memphis, TN 38163 USA; Department of Pathology, University of Tennessee Health Science Center, Memphis, TN 38163 USA

**Keywords:** Autoimmunity, Arthritis, Treg, Therapy, FTY720, Fingolimod, Type II collagen

## Abstract

**Background:**

Fingolimod (FTY720) is an immunomodulating drug that inhibits sphingosine-1-phosphate binding and blocks T-cell egress from lymph nodes. We analyzed the effect of FTY720 on the autoimmune T- and B-cell response in autoimmune arthritis and studied the mechanisms by which it alters the function of T cells.

**Methods:**

Human leukocyte antigen (HLA)-DR1 humanized mice were immunized with type II collagen (CII) and treated with FTY720 three times per week for 3 weeks. Arthritis was evaluated and autoimmune T- and B-cell responses were measured using proliferation assays, enzyme-linked immunosorbent assays, HLA-DR tetramers, and flow cytometry. The functional capacity of regulatory T (Treg) cells from FTY720-treated mice was measured using an in vitro suppression assay, and the role of Treg cells in inhibiting arthritis in FTY720-treated mice was evaluated using mice treated with anti-CD25 to deplete Treg cells.

**Results:**

Treatment with FTY720 delayed the onset of arthritis and significantly reduced disease incidence. FTY720 did not prevent the generation of a CII-specific autoimmune T-cell response in vivo. However, as the treatment continued, these T cells became unresponsive to restimulation with antigen in vitro, and this anergic state was reversed by addition of interleukin 2. Measurements of CD4^+^CD25^+^Foxp3^+^ cells in the lymph nodes revealed that the ratio of Treg to helper T (Th) cells increased twofold in the FTY720-treated mice, and in vitro assays indicated that the regulatory function of these cells was enhanced. That FTY720 stimulation of Treg cells played a major role in arthritis inhibition was demonstrated by a loss of disease inhibition and restitution of the T-cell proliferative function after in vivo depletion of the Treg cells.

**Conclusions:**

While FTY720 affects the recirculation of lymphocytes, its ability to inhibit the development of autoimmune arthritis involves several mechanisms, including the enhancement of Treg cell function by increasing the Treg/Th ratio and increased regulatory function on a per-cell basis. FTY720 did not inhibit the development of the autoimmune T-cell response, but disease inhibition appeared to be mediated by Treg cell–mediated suppression of the CII-specific T cells. These data suggest that specific targeting of Treg cells with FTY720 may be a novel therapy for autoimmunity.

**Electronic supplementary material:**

The online version of this article (doi:10.1186/s13075-015-0909-6) contains supplementary material, which is available to authorized users.

## Background

Autoimmunity is often considered a disease of immune imbalance between autoreactive helper T (Th) cells and regulatory T (Treg) cells. While thymic selection deletes the vast majority of autoreactive T cells during their maturation, a small but significant number of these autoreactive T cells survive thymic negative selection and reside in peripheral lymphoid tissue, where, under the right conditions, they can become activated and induce an autoimmune disease. Treg cells expressing the transcription factor Foxp3 play a major role in preventing activation and disease induction by these autoimmune Th cells, and maintaining a physiological balance between these two populations of cells is a major factor in preventing autoimmunity [1, 2]. Several investigators have demonstrated that when Treg cells are functionally deficient or absent in mice and humans, a variety of autoimmune disorders will develop [3–7]. Support for the role of Treg cells in preventing autoimmunity has been demonstrated in a number of studies where the administration of exogenously derived Treg cells inhibited the development of autoimmunity in several animal models [8–13]. Consequently, considerable efforts are being made to develop therapeutic means to inhibit autoimmune T-cell function by augmenting Treg cell numbers and/or their function.

One drug that has been studied for its ability to downregulate an autoimmune T-cell response is fingolimod (FTY720). FTY720 is an immunomodulatory drug that is structurally similar to sphingosine-1-phosphate (S1P), a lysophospholipid that affects a wide range of physiological activities, including lymphocyte trafficking and function [14, 15]. One of the immunomodulatory roles of S1P is its promotion of the egress of lymphocytes from the lymph nodes (LNs) to the bloodstream via the lymphatics [14]. Acting as an antagonist or partial agonist, FTY720 prevents S1P binding to its receptor and downregulates the receptor expression and signaling [16, 17]. The net effect is that lymphocyte recirculation from the LNs is blocked, causing lymphocytes to remain in the LNs and the peripheral blood to become lymphopenic [14]. As an immunomodulatory agent, FTY720 has been shown to have beneficial effects in several animal models in which T cells directly mediate the pathology, including experimental autoimmune encephalomyelitis (EAE) [18], diabetes [19], and inflammatory bowel disease [20]. However, it is less effective in other autoimmune disease models, such as systemic lupus erythematosus (SLE), where antibody predominates as the pathogenic mechanism [21]. Clinically, FTY720 has been approved as a treatment for relapsing multiple sclerosis [22, 23]. On the basis of its ability to inhibit the function of S1P promotion of lymphocyte recirculation, it was initially thought that the primary mechanism of autoimmune disease inhibition by FTY720 was the sequestration of T cells to secondary lymphoid organs, thereby preventing the movement of lymphocytes to the tissues targeted by the autoimmune T cells. However, recent studies have indicated that, in addition to sequestration, FTY720 treatment appears to drive down Th cell function and promote the activity and/or number of natural regulatory T (nTreg) cells [20, 24]. These observations have put a new focus on the mechanisms of FTY720 as a therapeutic agent of autoimmunity.

Using a human leukocyte antigen (HLA)-DR1 transgenic (Tg) mouse model of rheumatoid arthritis (RA), we investigated the ability of FTY720 to inhibit the development of autoimmune arthritis induced by immunization with type II collagen (CII). Given that disease manifestation in this model is dependent on an autoimmune T- and B-cell response, we sought to determine if FTY720 would be effective in preventing an autoimmune response where autoantibody plays a major role. Our studies indicate that while FTY720 inhibits the development of autoimmune disease in this model, its effect on B cells and T cells differs significantly. Our data indicate that FTY720 not only differentially modulates the migration of lymphocytes via the lymphatics but also enhances Treg function and alters the Th/Treg balance to a level sufficient to inhibit the function of autoimmune T cells and prevent the development of autoimmune arthritis.

## Methods

### Mice

The generation of DRB1*0101 Tg mice (DR1) has previously been described [25]. The human DRA1 and DRB1 transgenes were established in (C57BL/6 × SJL/J)F2 mice and backcrossed to the B10.M background, and the murine I-A^f^ molecule expressed by B10.M mice was genetically deleted by backcrossing the B6.129S2-*H2*^*dlAb1-Ea*^/J knockout (stock number 003584; The Jackson Laboratory, Bar Harbor, ME, USA) onto the B10.M-DR1 strain for six generations. A DR1 mouse expressing a CII-specific T-cell receptor (TCR) transgene was established using the cassette vectors pTαcass and pTβcass (gift of Dr. C. Benoist and Dr. D. Mathis, Harvard Medical School, Boston, MA, USA [26]). The rearranged T-cell receptor Vα2/Jα27 and Vβ8.1/Dβ1/Jβ2.4 fragments were derived from a DR1-restricted, CII_259–274_-specific T-cell hybridoma (E168) by polymerase chain reaction (PCR), cloned into PCR2.1, and confirmed by sequencing. These complementary DNA were subcloned into the expression vectors, generating pTαcass-Vα2 and pTβcass-Vβ8.1. The transgenes were established by injecting both genes into fertilized eggs of FVB/N mice. Tg founders were identified by PCR analysis of tail DNA, and transmission of the transgenes to subsequent generations was ascertained by PCR or flow cytometric analysis using peripheral blood lymphocytes. Mice were backcrossed onto the B10.M-DR1 mice to establish a B10.M-DR1-TCR Tg mouse strain. Mice used in these experiments were from generations N7–N9, and they were heterozygous for the TCR transgenes and homozygous for the HLA-DR1 transgene. All mice used in these studies were bred in our own facility, maintained in microisolators in a pathogen-free environment, and fed standard rodent chow (Nestlé Purina, St. Louis, MO, USA) and water ad libitum. All animal studies were approved by the Institutional Animal Care and Use Committee and the Research and Development Committee at the Memphis VA Medical Center, Memphis, TN, USA.

### Immunizations and arthritis

For arthritis induction and T-cell proliferation assays, 8- to 10-week-old mice were immunized subcutaneously at the base of the tail with 100 μg of bovine CII emulsified in complete Freund’s adjuvant (CFA) consisting of 85 % heavy paraffin oil (Fisher Scientific, Fair Lawn, NJ, USA), 15 % mannide monooleate (Sigma-Aldrich, St. Louis, MO, USA), and 4 mg/ml of heat-killed mycobacterium (Difco H37Ra; BD Diagnostics, Sparks, MD, USA) [27]. FTY720 (Cayman Chemical, Ann Arbor, MI, USA) was diluted in nitrogen-bubbled 10 % ethanol and injected intraperitoneally. Treatment schemes varied among experiments and are described in the Results section. In some experiments, mice were treated with 250 μg of rat anti-CD25 (clone PC61) or a control rat antibody (MOPC 167) once weekly for 3 weeks. Peripheral blood was monitored by flow cytometry to ensure efficacy in the depletion of the CD25^+^Foxp3^+^ T cells. For arthritis studies, mice were examined three times per week starting at day 18 after immunization, and the presence of arthritis, number of affected limbs, and severity of inflammation were assessed. Severity of disease was evaluated by visual inspection and assigned a score using a scale of 0–4 as described elsewhere [27].

### Measurement of antibody levels

CII-specific antibody levels in sera of mice immunized with CII/CFA were quantitated using a bead-based enzyme-linked immunosorbent assay and a Bio-Plex instrument (Bio-Rad Laboratories, Hercules, CA, USA). Fluorescent dye–labeled beads coupled with bovine CII were incubated with dilutions of mouse sera starting at 1:1000, and the quantity of bound mouse immunoglobulin G (IgG) was detected by the addition of a biotinylated anti-mouse IgG (Sigma-Aldrich) followed by phycoerythrin (PE)-streptavidin (Invitrogen, Carlsbad, CA, USA). The quantity of anti-CII antibody was calculated using a curve generated with purified CII-specific antibody as the standard.

### Antibodies and flow cytometry

Fluorescently labeled antibodies specific for CD3 (Alexa Fluor 700; Life Technologies, Carlsbad, CA, USA), CD4 (PE or Pacific Blue), CD8 (allophycocyanin [APC]), CD19 (peridinin chlorophyll-cyanine 5.5 [PerCP-Cy5.5]), CD25 (PerCP-Cy7), and CD62L (fluorescein isothiocyanate [FITC]) were obtained from BD Biosciences (San Jose, CA, USA). Intracellular staining for Foxp3 was performed using anti-mouse Foxp3 antibody labeled with FITC according to the manufacturer’s instructions (eBioscience, San Diego, CA, USA). Labeled cells were washed with phosphate-buffered saline (PBS), and a minimum of 10,000 cells were analyzed from each sample using a 5-laser (355, 405, 488, 561, and 640 nm) BD LSR II flow cytometer (BD Biosciences). To determine cell numbers, 50 μl of Molecular Probes CountBright beads (Thermo Fisher Scientific, Eugene, OR, USA) were added to each sample before cytometric analyses, and the number of cells per unit volume was calculated as (cell events/bead events) × (bead count/volume of blood sample analyzed). Data analysis was performed using FlowJo software (Tree Star, Ashland, OR, USA).

### Tetramer binding

CII-specific T cells from draining LNs were enumerated and characterized using a DR1-CII tetramer [28]. Briefly, soluble DR1 covalently linked to the immunodominant CII peptide was produced in S2 cells, affinity-purified, biotinylated by BirA (Avidity, Aurora, CO, USA), and formed into multimeric units by stepwise addition of PE-labeled streptavidin (Rockland Immunochemicals, Limerick, PA, USA). For identification of CII-specific T cells ex vivo, LN cells were incubated with 1 μg of the tetramer at 37 °C for 2.5 h in complete HL-1 medium (50 U/ml penicillin G sodium, 50 μg/ml streptomycin sulfate, 0.05 mM β-mercaptoethanol, 2 mM l-glutamine, and 0.1 % bovine serum albumin; BioWhittaker/Lonza, Walkersville, MD, USA) supplemented with 5 mM NaN_3_. At the end of the incubation, antibodies specific for various CD markers were added and cells were incubated for an additional 30 minutes at 4 °C. Samples were then washed and resuspended in PBS supplemented with 0.1 % NaN_3_ and 2 % fetal bovine serum and analyzed by flow cytometry (BD LSR II). DR1-restricted, CII_257–274_-specific and HA_306–318_-specific T-cell hybridomas were used as positive and negative controls, respectively, to monitor the specificity and sensitivity of the DR1-CII tetramer (see Additional file 1).

### T-cell proliferation and Treg assays

Total lymphocytes or CD4^+^CD25^−^ T cells were recovered from draining LNs of CII-immunized mice treated with FTY720 or vehicle and cultured in the presence of the CII_257–274_ peptide and antigen-presenting cells for 4 days. T-cell proliferation assays were performed in 96-well microtiter plates in a total volume of 0.3 ml containing 4 × 10^5^ LN cells and various concentrations of the CII_257–274_ peptide in complete HL-1 medium. For experiments using purified CD4^+^ T cells, equal numbers of naive spleen cells were added as antigen-presenting cells. Cell cultures were maintained at 37 °C in 5 % humidified CO_2_ for 4 days. On day 3 of culture, 1 μCi of [^3^H]thymidine was added, and on day 4 the plates were harvested onto filter plates. After the filter plates were dried, scintillation fluid was added and [^3^H]thymidine incorporation was measured using a TopCount NXT instrument (PerkinElmer, Waltham, MA, USA).

Treg cell assays were performed using Treg cells purified with an autoMACS separator (Miltenyi Biotec, San Diego, CA, USA), and CII-specific T cells as responders were recovered from the B10.M-DR1-TCR Tg mouse. Treg cells were purified from LN cells of mice treated with FTY720 or vehicle by negative selection for CD4^+^ T cells and positive selection for CD25^+^ T cells (Miltenyi Biotec). The quality of the purification was monitored using antibodies specific for CD4, CD25, and Foxp3 and by flow cytometric analysis (BD LSR II). The negative selection resulted in ≥85 % enrichment of CD4^+^ cells, and positive selection yielded CD4^+^CD25^+^ cells at ≥95 %. Splenocytes containing Tg CII-specific T cells were used as the responders in the Treg assays and were labeled with 2.5 μM CellTrace Violet as described by the manufacturer (Invitrogen) before their addition to the Treg assays. The Treg cells and the labeled T responders were cocultured in 96-well plates at ratios of 1:1 to 1:16 Treg/T responder cells in the presence of 10 μg of CII_257–274_ peptide. After 3 days of culture, cells were recovered from the cultures; stained with 4′,6-diamidino-2-phenylindole (DAPI; Sigma-Aldrich) and antibodies specific for CD19 (PerCP-Cy5.5), CD8-APC, CD4-PE, and CD3-Alexa Fluor 700 (BD Biosciences); and analyzed by flow cytometry. Our data analysis was based on sequential gating of live cells (DAPI-negative), low angle versus side scatter, CD8^−^ and CD19^−^, CD3^+^, and CD4^+^ cells. Generations of T-cell division were determined by generational dilution of the CellTrace Violet dye, and gates were drawn to determine the percentage of responder T cells that were in cellular division.

Cell-mixing proliferation experiments were performed as described above using purified CII-specific T cells and antigen-presenting cells from LN cells of mice treated with FTY720 or vehicle. The DR1-TCR Tg mouse was used as the source of CII T cells. The T cells were purified using an autoMACS instrument as described above. The antigen-presenting cell population was collected as a negative selection population following T-cell depletion. All purifications were monitored for quality by flow cytometry to ensure depletion of Treg cells, positive selection of CD4^+^CD25^−^ cells, and enrichment for antigen-presenting cells. The purified antigen-presenting cell and T-cell populations were recombined in culture in all possible permutations and stimulated with CII peptide, and proliferation was measured by [^3^H]thymidine incorporation as described above.

## Results

### FTY720 inhibits the development of autoimmune arthritis

**Fig. 1:**
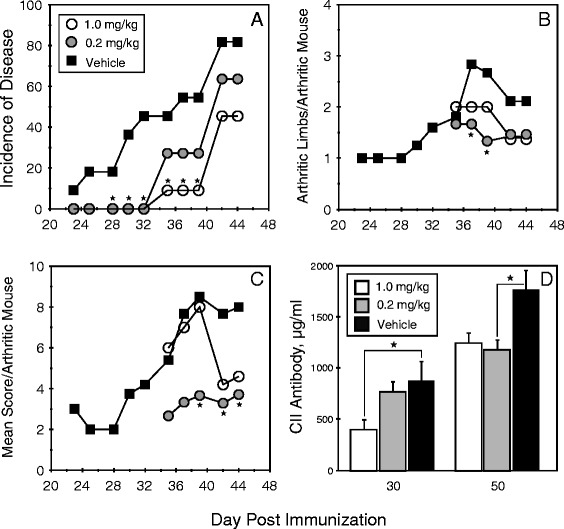
Treatment of mice with FTY720 reduces incidence and severity of autoimmune arthritis. DR1 transgenic mice were immunized subcutaneously with 100 μg of type II collagen (CII) emulsified in complete Freund’s adjuvant, and they were given intraperitoneal injections of FTY720 or vehicle control three times per week for 3 weeks starting on the day of immunization (day 0), for a total of nine injections. **a** FTY720 at 1.0 mg/kg (*open circles*) and 0.2 mg/kg (*gray circles*) delayed the onset of disease (mean day 33 for control and days 40 and 42 for FTY720 groups; *p* < 0.03 for both groups) and reduced the disease incidence (*p* < 0.05 for days 30 through 40 for 1 mg/kg group, indicated by *closed stars*) in comparison to the vehicle control group (*closed circles*; *n* = 11 for each group). **b** Both doses of FTY720 reduced the number of arthritic limbs observed per arthritic mouse. *Closed stars* indicate *p* < 0.05. **c** Both doses of FTY720 reduced the mean arthritic score per arthritic mouse. *Closed stars* indicate *p* < 0.05. **d** FTY720 treatment reduced the production of CII-specific autoantibody. Antibody concentrations in sera obtained from the mice at days 30 and 50 after immunization were measured using a bead-based enzyme-linked immunosorbent assay, and antibody concentrations were calculated using a CII-specific antibody standard. *Closed stars* indicate *p* < 0.05; error bars indicate standard deviation

To determine the effect of FTY720 on the development of autoimmune arthritis, DR1 Tg mice immunized with CII/CFA were treated with 1 mg/kg or 0.2 mg/kg of FTY720 every 3 days for a total of nine treatments over 3 weeks, and arthritis incidence and severity were evaluated. As shown in Fig. 1, both doses of FTY720 significantly inhibited arthritis development. Animals receiving FTY720 had reduced incidence and delayed onset of disease (Fig. 1a), and the severity of disease was also significantly reduced (Fig. 1b and c). The mean times of onset of disease for the FTY720 groups were day 42 and day 40 (1 mg/kg and 0.2 mg/kg, respectively), as opposed to day 33 for the control group (*p* < 0.03 for both groups), and FTY720 treatment decreased the incidence of arthritis by as much as 50 % (1 mg/kg) (Fig. 1a). The severity of disease in terms of arthritic limbs per arthritic mouse and mean severity score per arthritic mouse was also significantly decreased in animals that received the drug (Fig. 1b and c). The three initial time points for the 1 mg/kg group depicted in Fig. 1b and c (days 35, 37, and 39) were based on only one arthritic mouse and two limbs at each of these time points, making comparison of these early evaluations of severity less reliable. As the incidence of arthritis increased in the 1 mg/kg group at the later time points, the mean severity of disease per mouse dropped to 50 % of that observed in the control group. While CII-specific autoantibody concentrations were also significantly lower in the FTY720-treated mice (Fig. 1d), appreciable concentrations of autoantibody were detected in both FTY720 groups at both time points. These data are consistent with the arthritis incidence data (Fig. 1a) in that while arthritis was delayed in the FTY720 groups, some mice eventually developed disease.

### FTY720 alters the number and subpopulation distribution of B and T cells in the peripheral blood and lymph nodes

**Fig. 2:**
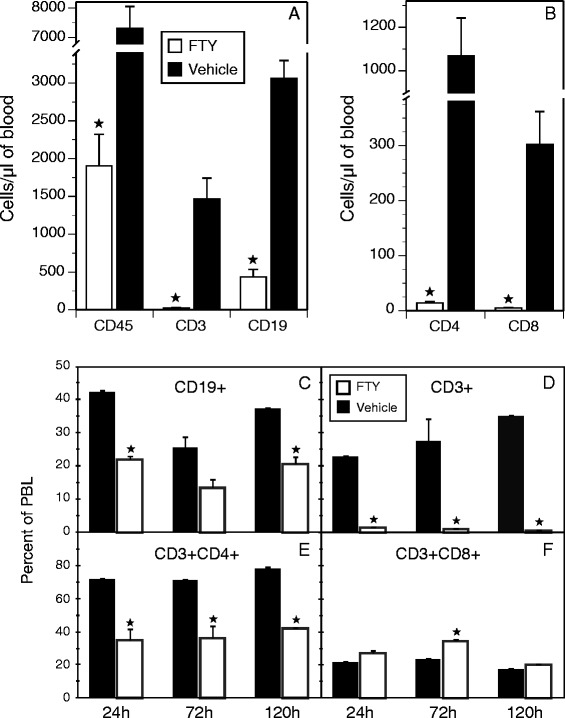
FTY720 (FTY) treatment alters the number and ratio of lymphocyte populations in the peripheral blood of type II collagen–immunized mice. Peripheral blood lymphocytes (PBLs) were recovered from mice treated with FTY720 at 24 h, 72 h, and 120 h after a single 1 mg/kg treatment, and the cells were stained with antibodies specific for CD19, CD3, CD4, and CD8. All data were gated on the basis of live cells (4′,6-diamidino-2-phenylindole–negative) and forward versus side scatter to identify the lymphoid population. The CD4^+^ and CD8^+^ populations were generated as subpopulations of CD3^+^ cells. **a** and **b** The number of cells per microliter of blood was determined by adding CountBright beads to the samples before analyses, as described in the Methods section. Closed stars in (**a**) indicate statistically significant changes (*p* < 0.01). **c**–**f** Change in the subpopulation distribution of B cells and T cells in the peripheral blood. Antibody staining and analysis of the flow cytometry data were done as indicated in each panel, with statistically significant differences of *p* < 0.05 denoted by *closed stars. Open bars* indicate 1.0 mg/kg FTY720 treatment, and *closed bars* represent vehicle control. Error bars depict standard deviations

To determine how the autoimmune response was affected by FTY720, peripheral blood and draining LNs from mice treated with the drug or control vehicle were analyzed for lymphocyte numbers and composition. As reported elsewhere [14, 29, 30], treatment of mice with FTY720 greatly reduced the number of B and T lymphocytes in the peripheral blood (22 % and <2 % of levels in control mice, respectively), but it also altered the lymphocyte subset distributions (Fig. 2). After 24 h, the total number of circulating B and T cells dropped dramatically (Fig. 2a) and the number of CD3^+^CD4^+^ and CD3^+^CD8^+^ cells were similarly decreased (Fig. 2b). The composition of the lymphocyte subpopulations that remained in the circulation of the FTY720-treated mice was altered compared with the vehicle control group. While the percentage of CD19^+^ B cells in the total lymphocyte population in the peripheral blood dropped by 50 % after treatment with FTY720 (Fig. 2c), the CD3^+^ T-cell population was reduced to <5 % of levels found in vehicle-treated mice (Fig. 2d). These changes were primarily the result of decreases in the CD4^+^ subpopulation; the percentage of CD8^+^ T cells was relatively unchanged (Fig. 2e and f). Thus, while FTY720 induced a significant decrease in the total number of lymphocytes in the peripheral blood, it also altered the CD4/CD8 T-cell ratio.

**Fig. 3:**
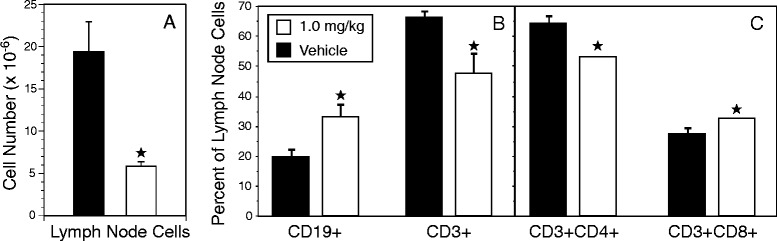
FTY720 treatment alters the total cell number and ratio of lymphocyte populations in the draining lymph nodes (LNs) of type II collagen–immunized mice. Cells were recovered from draining LNs after immunization and nine treatments with FTY720 as described in the Fig. 1 legend. The number of cells in the LNs of FTY720-treated mice was significantly lower than the number found in the LNs of controls (**a**). The ratios of B-cell and T-cell subpopulations in the LNs (**b** and **c**) were statistically different between FTY720-treated and control mice, although the differences were significantly less than the differences observed in the peripheral blood. Antibody staining, measurement of cell numbers, and data analysis were performed as described in the Fig. 2 legend. *Closed stars* indicate statistically significant differences (*p* < 0.05). *Open bars* indicate 1.0 mg/kg FTY720 treatment, and *closed bars* represent vehicle control. Error bars indicate standard deviation

While one of the known functions of FTY720 is to block the recirculation of lymphocytes from the LNs to the blood by preventing the binding of S1P [31], we found no concomitant increase in the number of lymphocytes in the draining LNs of the drug-treated mice (Fig. 3). As in the peripheral blood, LNs from immunized mice treated with FTY720 contained fewer total lymphocytes than LNs from immunized mice treated with vehicle (Fig. 3a), and the subpopulation distribution of lymphocytes was also altered (Fig. 3b and c). FTY720 treatment resulted in an increase in the percentage of CD19^+^ cells and a decrease in the percentage of CD3^+^ cells (Fig. 3b), leading to a change in the CD3/CD19 ratio from 3.2:1 in vehicle-treated mice to 1.2:1 in FTY720-treated mice. Similarly, the ratio of CD4^+^ to CD8^+^ T cells was also altered, but not to the degree observed in the peripheral blood. The percentage of CD3^+^CD4^+^ cells decreased from 65 % in the vehicle group to 53 % of the total CD3^+^ cells in the treated mice, and the CD3^+^CD8^+^ cells increased marginally, from 27 % to 34 % of the CD3^+^ cells (Fig. 3c).

### FTY720 does not inhibit the generation of the autoreactive T-cell response in vivo

**Fig. 4:**
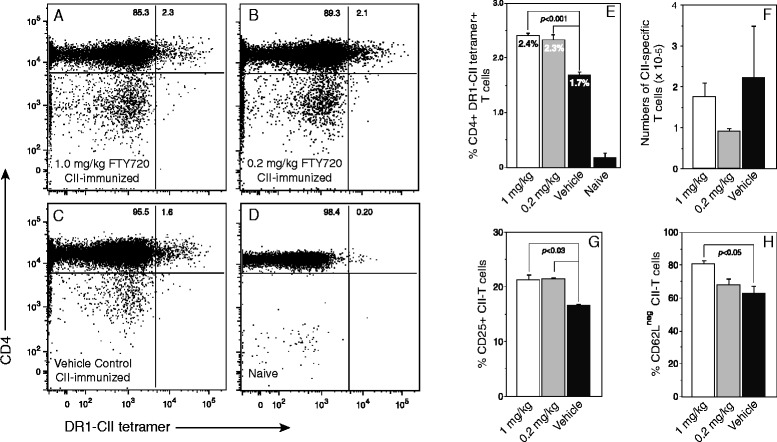
Ex vivo analysis of the effect of FTY720 on the type II collagen (CII)-specific T-cell response. Mice were immunized with CII and complete Freund’s adjuvant and treated with FTY720 (1.0 mg/kg or 0.2 mg/kg) or vehicle, or they were not immunized and not treated (naive). Lymph node cells recovered from the mice treated with FTY720 or vehicle (**a**–**c**) or from naive mice (**d**) were stained with a DR1-CII tetramer and antibodies specific for CD19, CD3, CD4, CD8, CD25, and CD62L, and the cells were analyzed by flow cytometry. The mean percentage of CD4^+^/tetramer-positive, CII-specific T cells in the lymph nodes is shown in (**e**), and the number of CII-specific T cells present in the lymph nodes as determined by tetramer staining and cell counts is shown in (**f**). The expression of CD25 and CD62L by the tetramer-positive cells in the FTY720 and control groups as determined by flow cytometry is shown in (**g**) and (**h**). Data were generated by sequential gating on 4′,6-diamidino-2-phenylindole–negative cells, forward versus side scatter, CD19^−^ and CD8^−^ cells, and CD3^+^CD4^+^ cells. The tetramer-positive cells in (**a**–**e**) are shown as the percentage of CD3^+^CD4^+^ T cells, and this percentage value was used to calculate the total number of CII-specific T cells shown in (**f**). *Open bars* indicate mice treated with 1.0 mg/kg FTY720, *gray bars* represent mice treated with 0.2 mg/kg, and *closed bars* indicate the vehicle control group. Error bars indicate standard deviation, and *p* values indicate statistically significant differences between treatment groups and vehicle groups

Because FTY720 has a strong effect on CD4^+^ T-cell recirculation, and because collagen-induced arthritis is mediated by CII-specific CD4^+^ T cells [28], we investigated how FTY720 affected the development of the CD4^+^ CII-specific autoimmune T-cell response in the DR1 Tg mice (Fig. 4). LN cells were recovered from CII-immunized mice treated with FTY720 or vehicle as described for Fig. 1, as well as in unimmunized mice, and the number of CII-specific T cells present was determined using a PE-labeled DR1-CII tetramer in combination with fluorescently labeled antibodies specific for several CD markers. Data analysis was focused on live cells using DAPI exclusion, CD19^−^ and CD8^−^ cells, and CD3^+^CD4^+^, DR1-CII tetramer-positive cells (Fig. 4a–d). FTY720 did not inhibit the development of the CD4^+^ CII-specific T-cell response. In fact, the percentages of CII T cells in both the 1.0 mg/kg and 0.2 mg/kg FTY720-treated groups were statistically higher than those found in the vehicle-treated control group (Fig. 4e). However, due to the smaller total number of cells in the LNs from FTY720-treated mice (Fig. 3a), the total number of CII-specific T cells found in the LNs were not statistically different between these groups (Fig. 4f). Analysis of activation markers expressed by the tetramer-positive, CII-specific T cells indicated that more of the cells from the FTY720-treated mice expressed CD25 and fewer expressed CD62L in comparison to the control group (Fig. 4g and h), both indications that these T cells were stimulated by antigen in vivo.

### CII-specific T cells from FTY720-treated mice are anergic to restimulation in vitro

**Fig. 5:**
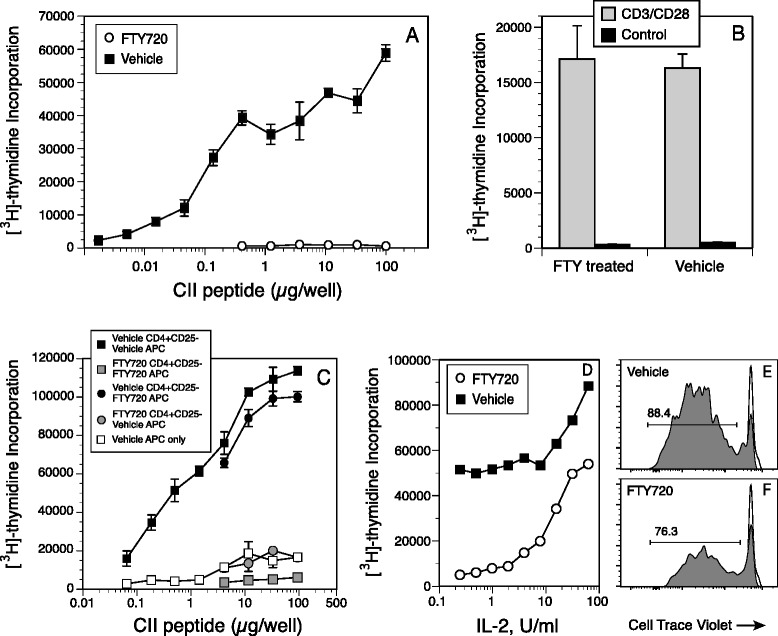
Proliferative response of type II collagen (CII)-specific T cells recovered from mice treated with FTY720 (FTY). Total lymphocytes or CD4^+^CD25^−^ T cells were recovered from draining lymph nodes of CII-immunized mice treated with 1 mg/kg of FTY720 or vehicle and cultured in the presence of the CII_257–274_ peptide for 4 days. [^3^H]thymidine was added to the cultures on day 3, and cells were harvested on the following day. Controls were unstimulated cells. Error bars indicate standard deviation. **a** In vitro proliferative response of lymph node cells from DR1 transgenic mice immunized with CII and treated with 1.0 mg/kg FTY720 or vehicle three times per week for 3 weeks. **b** Lymph node cells from mice treated with 1.0 mg/kg FTY720 or vehicle were stimulated in vitro with anti-CD3/anti-CD28 antibodies (Ab). **c** Proliferative response of CD4^+^CD25^−^ lymph node cells from CII-immunized mice treated with 1.0 mg/kg FTY720 or vehicle. CD4^+^CD25^−^ T cells were purified using magnetic bead separation and mixed with antigen-presenting cells depleted of CD4^+^ cells recovered from the spleens of FTY720- or vehicle-treated mice. **d** Addition of interleukin (IL)-2 to cultures of T cells from mice treated with FTY720 restored the CII-stimulated proliferative response. **e** and **f** DR1-CII tetramer analyses of the proliferative response shown in (**d**). Lymph node cells from FTY720- or vehicle-treated mice immunized with CII were labeled with CellTrace Violet, placed in culture, and stimulated with the CII peptide. After 3 days, cells were recovered and stained with DR1-CII tetramer; antibodies to CD3, CD4, and CD8; and 4′,6-diamidino-2-phenylindole (DAPI). Data shown are the percentages of CD4^+^ DR1-CII tetramer-positive cells in cellular division as indicated by the loss of CellTrace Violet fluorescence. Data were sequentially gated on DAPI^−^, CD3^+^, and CD8^−^ cells. *Gray histograms* are the CD4^+^ DR1-CII tetramer-positive cells, and *open histograms* are derived from CellTrace Violet–labeled cells that were not stimulated to divide

Despite the generation of a CII-specific T-cell response in the draining LNs of immunized mice treated with FTY720, these T cells were totally refractory to restimulation in vitro (Fig. 5). Mice were immunized and treated with FTY720 as described in the Fig. 1 legend, and LN cells were collected on day 18 and stimulated with the CII peptide CII_257–275_ that contains the immunodominant determinant for HLA-DR1 [25]. As depicted in Fig. 5a, the T cells from LNs of control mice responded strongly to peptide stimulation, while the T cells of the FTY720-treated mice were completely unresponsive to stimulation at all concentrations of the CII peptide tested. FTY720 did not induce a general unresponsiveness in T cells, as stimulation of T cells from FTY720-treated mice with anti-CD3 and anti-CD28 antibodies generated a proliferative response that was indistinguishable from that observed with T cells from control mice (Fig. 5b).

Cell-mixing experiments were performed to determine if the CII-specific T cells were anergic or if FTY720-treated antigen-presenting cells or Treg cells were suppressing or preventing their response in vitro. Antigen-presenting cells and CD4^+^CD25^−^ CII-specific T-cell populations from FTY720- and vehicle-treated DR1-TCR Tg mice were purified with magnetic beads, recombined in culture in all possible permutations, and stimulated with the CII peptide. This approach effectively removed Treg cells from the cultures and allowed us to determine if FTY720 altered the function of the antigen-presenting cells or if the T cells were rendered unresponsive in vivo. Regardless of whether the source of antigen-presenting cells was from FTY720-treated mice or vehicle-treated mice, the CII-specific T cells from FTY720-treated mice were totally unresponsive to stimulation by antigen-presenting cell presentation of the CII peptide (Fig. 5c). Additionally, antigen-presenting cells from FTY720-treated mice were fully capable of stimulating CII-specific T cells from control mice. These data indicate that the antigen-presenting cells from FTY720-treated mice were not preventing the antigen-specific stimulation of the CII-specific T cells, and also that the CII-specific T cells from the FTY720-treated mice were rendered functionally unresponsive to restimulation in vivo. Because these experiments were based on the response of identical numbers of CD4^+^ T cells from both groups, these data also indicate that the percentage decrease in CD4^+^ T cells observed in the LNs of FTY720-treated mice (Fig. 3) was not responsible for the diminished proliferative response (Fig. 5a).

To determine if the CII-specific T cells from the FTY720-treated mice were anergic, CII-specific proliferative assays were performed in the presence of various concentrations of exogenous interleukin (IL)-2. As shown in Fig. 5d–f, the addition of IL-2 to these cultures restored the proliferative response of the CII-specific T cells from the FTY720-treated mice. The FTY720 T cells responded in a dose-restricted manner, and their proliferative response reached levels similar to levels in control T cells stimulated with the CII peptide alone (Fig. 5a). These data are supported by tetramer analysis of CellTrace Violet–labeled cells (Fig. 5e and f). LN cells recovered from FTY720- and vehicle-treated mice were labeled with CellTrace Violet and stimulated in culture with IL-2 and CII peptide. After 3 days, cells were recovered and stained with DR1-CII tetramer and antibodies to CD3, CD4, and CD8 and analyzed by flow cytometry. As shown in Fig. 5f, the addition of IL-2 to the CII-stimulated cultures of lymphocytes from the FTY720-treated mice generated a population of T cells that bound the DR1-CII tetramer, and >75 % of these cells were in division, as indicated by the dilution of the CellTrace Violet dye. These data are similar to those observed with IL-2, CII-stimulated T cells from the vehicle control mice (Fig. 5e), in which 88 % of the CD4 ^+^ DR1-CII tetramer-positive cells were in division. These data support the concept that FTY720-mediated inhibition of the development of autoimmune arthritis in this model is mediated by an in vivo induction of anergy in the autoimmune T cells.

### Elevated ratios in vivo and enhanced function of Foxp3^+^ Treg cells from FTY720-treated mice

**Fig. 6:**
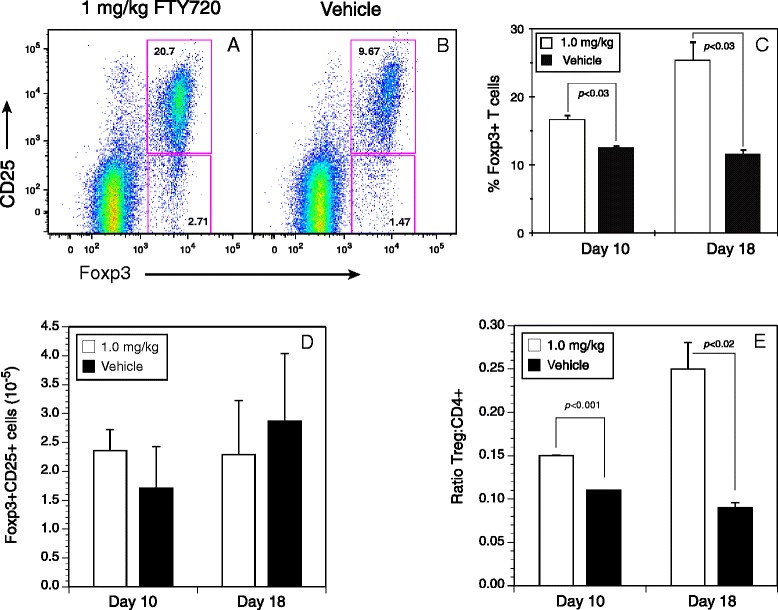
The ratio of regulatory T (Treg) cells to helper T cells is increased in lymph nodes of FTY720-treated mice. Lymph node cells from DR1 transgenic mice immunized with type II collagen and treated with FTY720 or vehicle three times per week (total of eight doses) were recovered on day 17 and stained with antibodies specific for CD4, CD8, CD19, CD3, CD25, and Foxp3, and they were analyzed by flow cytometry. **a** and **b** Representative scatterplots (1 mg/kg FTY720 and vehicle control, respectively) of data from at least three repeat experiments, summarized in (**c**). *Open bars* indicate 1.0 mg/kg FTY720, and *closed bars* represent 1.0 mg/kg vehicle control. The number of Treg cells per lymph node shown in (**d**) was determined using CountBright beads as described in the Methods section. The differences observed were not statistically significant. The ratio of Treg cells to CD4^+^ cells shown in (**e**) was calculated by dividing the number of both cell populations measured using the CountBright beads. Flow cytometry data shown were derived from sequential gating on live cells (4′,6-diamidino-2-phenylindole–negative), forward versus side scatter, CD8^−^ and CD19^−^, CD3^+^, and CD4^+^ cells. Error bars indicate standard deviation

Because the CII-specific T cells from FTY720-treated mice were unresponsive to antigen stimulation in vitro, we sought to determine if the number or ratio of Treg cells in FTY720-treated mice was altered in comparison to the control mice. At days 10 and 18 after immunization and FTY720 treatment (four doses of drug for day 10 mice, eight doses for day 18), draining LNs were recovered and the percentage of CD4^+^CD25^+^Foxp3^+^ Treg cells present was determined by flow cytometry. Data were analyzed by gating on CD19^−^ and CD3^+^CD4^+^ cells, and percentages were calculated on CD4^+^CD25^+^Foxp3^+^ cells (Fig. 6a and b). As shown in Fig. 6c, 10 days after FTY720 treatment, there was a 40 % increase in the percentage of CD25^+^Foxp3^+^ Treg cells in the LNs of these mice in comparison to the vehicle-treated group, and by day 18 there was a 100 % increase. While the drug did not induce a significant increase in the number of Treg cells in the LNs of these mice (Fig. 6d), it did induce a significant increase in the Treg/Th ratio in these lymphoid organs (Fig. 6e).

**Fig. 7:**
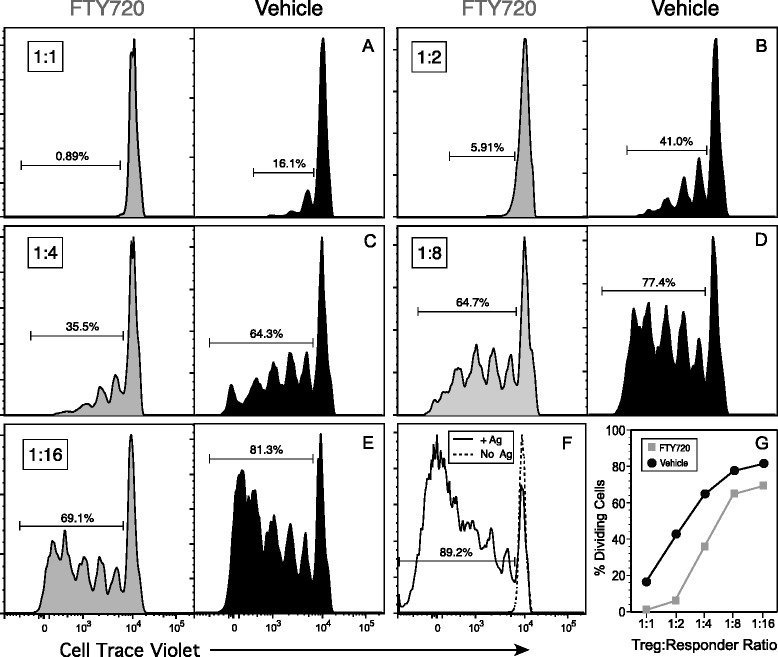
Functional analysis of the regulatory T (Treg) cells recovered from mice treated with FTY720. DR1 transgenic mice were immunized with type II collagen (CII) and treated three times per week (total of eight doses) with 1.0 mg/kg of FTY720 or vehicle. On day 17, CD4^+^CD25^+^ Treg cells were recovered from the spleens of these mice using magnetic bead selection, and they were tested for their ability to inhibit a CII-specific T-cell proliferative response. **a**–**e** Responder T cells from naive mice were labeled with CellTrace Violet and cocultured with the Treg cells at ratios ranging from 1:1 to 1:16 (Treg/responder) in the presence of 10 μg of CII_257–274_ peptide. After 3 days of culture, cells were collected and stained with antibodies specific for CD3, CD4, CD8, and CD19 and analyzed by flow cytometry. Data shown are based on sequential gating of live cells (4′,6-diamidino-2-phenylindole–negative), forward versus side scatter, CD8^−^ and CD19^−^, CD3^+^, and CD4^+^ cells. Histograms indicate generation of T-cell division by dilution of the CellTrace Violet label, and gates indicate percentage of responder T cells that were in division. **f** T-cell proliferation with peptide added and no Treg cells added (positive control, *solid line*), and T cells with no Treg cells and no peptide (negative control, *dotted line*). **g** Summary of percentage of cells dividing at each of the responder/Treg ratios. Data are representative of three independent experiments

**Fig. 8:**
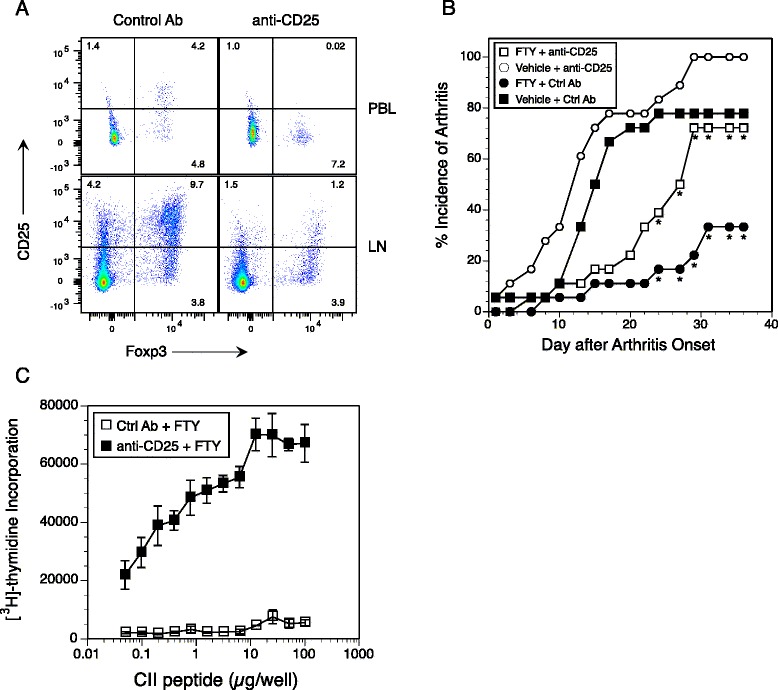
Depletion of CD25^+^ regulatory T (Treg) cells reduces the inhibitory effect of FTY720 on the development of autoimmune arthritis and restores the type II collagen (CII)-specific T-cell response. Mice were immunized with CII on day 0 and split into four groups for treatment: (1) FTY720 + anti-CD25 antibody (Ab), (2) vehicle + anti-CD25 antibody, (3) FTY720 + control antibody (MOPC 167), and (4) vehicle + control antibody. Two days before immunization with CII, mice were treated with 250 μg of antibody (anti-CD25 or control), and antibody treatments were continued for 3 weeks with one treatment per week. **a** Representative data show that anti-CD25 treatment in the Treg cell population significantly depleted the CD25^+^Foxp3^+^ population in the peripheral blood and lymph nodes, and also significantly reduced the number of Foxp3^+^ cells in the lymph nodes. Starting at the time of immunization, mice were treated with FTY720, and treatment was continued three times per week for a total of nine doses. **b** Mice were scored three times per week for arthritis starting on day 18 after immunization. Data shown were derived from two separate experiments and normalized to the first day of disease onset for each experiment. The *asterisks* indicate statistically significant differences in arthritis incidence between the FTY720 + anti-CD25 group and the FTY720 + control Ab group in the study (*p* < 0.04 by χ^2^ test). **c** CII-specific proliferative response of T cells from the mice presented in (**b**). On day 17 after immunization, cells from draining lymph nodes were placed in culture and restimulated with the CII_257–274_ peptide. Proliferation was measured by [^3^H]thymidine incorporation, and data are representative of two experiments. Error bars indicate standard deviation

Given the increased Treg/Th ratio in the mice treated with FTY720, we evaluated the functional capacity of these Treg cells to inhibit a CII-specific T-cell proliferative response in vitro. Treg cells enriched by a combination of negative selection for CD4^+^ T cells and positive selection for CD25^+^ cells using magnetic beads were cultured at various ratios with CII-specific T cells labeled with CellTrace Violet. After 3 days, cells were recovered; labeled with fluorescent antibodies specific for CD19, CD3, CD8, and CD4; and analyzed by flow cytometry (Fig. 7). The data shown in Fig. 7 represent the violet fluorochrome fluorescence intensity of the CD3^+^CD4^+^ cells, and the gates indicate the percentage of cells in division. Figure 7f shows the proliferative response of the CII-specific T cells in the absence of Treg cells where 89.2 % of the cells were in division. At the Treg/Th ratio of 1:1, the Treg cells from the FTY720-treated mice completely shut down the proliferative response of the CII-specific T cells (Fig. 7a, *left panel*), while 16 % of the CII-specific T cells were in division in the presence of the Treg cells from the control mice (Fig. 7a, *right panel*). Similarly, at each of the ratios tested through 1:16 (Fig. 8b–e), the Treg cells from the FTY720-treated mice had significantly more regulatory capacity on a per-cell basis than the Treg cells from the control mice (Fig. 7g). The dose effect of the Treg cells was clearly evident in both groups, but the Treg cells from the FTY720 group always had a stronger regulatory effect on the CII-specific T-cell response. Thus, not only was the ratio of Treg/Th cells higher in the FTY720 mice but also, on a per-cell basis, the FTY720-derived Treg cells had an enhanced capacity to inhibit the CII-specific CD4^+^ T-cell responses.

To test the hypothesis that enhancement of Treg activity by FTY720 plays a role in the inhibition of autoimmune arthritis (depicted in Fig. 1), arthritis studies were performed with mice depleted of Treg cells and treated with FTY720. Two days before arthritis induction, mice were treated with 250 μg of anti-CD25 (clone PC61) or control antibody (MOPC 167) once weekly for 3 weeks. To monitor the efficacy of the antibody treatment, peripheral blood samples were collected 3 days after the first treatment and were found to be depleted of CD4^+^CD25^+^Foxp3^+^ cells (Fig. 8a). Similarly, LNs were significantly depleted of CD4^+^CD25^+^Foxp3^+^ Treg cells, and the few Foxp3^+^ cells that remained were CD25^−^ (Fig. 8a, *bottom panels*). Starting at the time of immunization, mice were treated three times per week with FTY720 or vehicle as described in the Fig. 1 legend. As shown in Fig. 8b, mice treated only with anti-CD25 developed arthritis earlier, at a higher incidence, and with a more severe form of the disease in comparison to all groups tested. The data depicted in Fig. 8b are a summation of two independent experiments, and they are synchronized to the first day on which arthritis was observed in each experiment. Similarly to the data shown in Fig. 1, treatment with FTY720 again caused a significant delay in the onset (mean 49.6 days vs. 33.2 days for vehicle control; *p* < 0.015) and a significantly lower incidence of disease in comparison to the control group (vehicle + control antibody; *p* < 0.01). In comparison, mice that received anti-CD25 and FTY720 developed a significantly higher incidence of disease (*p* < 0.04 at days 24–60) and an earlier disease onset (day 39.0; *p* < 0.056) than those treated with FTY720 alone, indicating that the depletion of Treg cells significantly reduced the inhibitory effect of FTY720 on arthritis development. The anti-CD25 treatment not only abrogated the effect of the FTY720 on arthritis development but also restored the proliferative response of the CII-specific T cells from these mice. As shown in Fig. 8c, T cells recovered from the draining LNs of mice treated with FTY720 and anti-CD25 had strong proliferative responses following stimulation with the CII peptide, while T cells from the FTY720-treated mice were again totally unresponsive. These data support the concept that at least one mechanism of FTY720 inhibition of arthritis development in this model involves enhancement of Treg function that downregulates the autoimmune T-cell response.

## Discussion

Earlier studies of the use of FTY720 as a therapeutic agent for autoimmunity were focused primarily on disease models in which the pathogenesis is mediated directly by T cells [18–20, 32]. In the present study, we investigated the effect of FTY720 on a model of autoimmune arthritis that requires both T and B cells for pathogenesis, and we examined how the function of these cells is affected by this drug. Treatment of the DR1 Tg mice with FTY720 resulted in a dose-dependent inhibition of the incidence and severity of arthritis concomitant with a reduced production of pathogenic CII-specific antibody. While FTY720 alters the recirculation of lymphocytes from the LNs to the bloodstream [14, 29, 30], our data indicate that it had a greater effect on CD3^+^ cells and a more pronounced effect on the CD4^+^ subpopulation. Though FTY720 inhibited the development of arthritis, surprisingly it did not prevent the generation of the CII-specific autoimmune T-cell response. CII-specific T cells were present at a higher percentage of CD4 cells in the LNs of FTY720-treated mice compared with controls, but in similar total numbers, and they expressed markers of activation indicating that they had been stimulated by antigen in vivo. Functionally, however, these T cells were unresponsive to restimulation with antigen in vitro, an observation consistent with the lower autoantibody levels and decreased incidence of disease. That these T cells were anergic was demonstrated by the reversal of their unresponsive state by the addition of IL-2. The unresponsiveness of the autoimmune T cells was coincident with an increase in the Treg/Th cell ratio in vivo. Treg cells in the LNs of the FTY720-treated mice constituted twice the normal percentage found in control mice, and they were found to be significantly more effective on a per-cell basis in regulating Th-cell responses. That Treg cells were playing a central role in the FTY720-mediated inhibition of arthritis was supported by depletion of Treg cells in vivo and the concomitant loss of the protective effect of FTY720 against disease and restoration of their recall proliferative response. Thus, these data indicate that a primary mechanism by which FTY720 inhibits arthritis in this model is through its potentiation of Treg-cell activity that downregulates the function of autoimmune T cells.

FTY720 has been shown to be effective as an immunomodulatory agent to varying degrees in several models of autoimmune disease, including EAE, diabetes, SLE, adjuvant arthritis, and inflammatory bowel disease [18–21, 32]. Similarly to many of these studies, treatment of mice with FTY720 in our autoimmune arthritis model did not completely prevent autoimmune arthritis from developing. Although the data in Fig. 1 were based on only nine treatments following the induction of the autoimmune response, continuous administration of FTY720 for the duration of the experiment was no more effective than treatment through only the first 21 days (data not shown). In both cases, disease was delayed and the incidence was reduced, but some mice still generated an autoimmune response and developed the disease. However, in all cases, disease severity was decreased.

Initially, the primary mechanism by which FTY720 inhibits the development of autoimmunity was thought to be based on its ability to prevent the recirculation of T cells. FTY720 is a structural analogue of S1P. S1P mediates lymphocyte recirculation by inducing the egress of lymphocytes from the LNs to eventually enter the peripheral bloodstream. In vivo FTY720 becomes phosphorylated and binds to four of the five S1P receptors (S1PRs), with its affinity for the S1P1R being the highest [31, 33]. The initial effect is partial agonist activation of the S1PR, followed by their internalization. Unable to use S1P signaling, the T cells exposed to FTY720 become trapped in the LNs, and thus it was suspected that disease inhibition was a result of preventing the movement of the pathogenic lymphocytes to the sites of inflammation. Our data indicate that FTY720 differentially inhibits the recirculation of B cells and subpopulations of T cells from the LNs to the peripheral blood, with its greatest effect being on CD4^+^ T cells and its least effect being on B cells. As expected, the total number of lymphocytes in the blood was significantly reduced, but T cells were affected more than B cells and CD4 cells more than CD8 cells. Studies by others indicate that the small numbers of T cells that remain in the peripheral blood of FTY720-treated mice are predominantly activated and memory cells [19]. Changes in lymphocyte composition were less dramatic in the LNs where an increase in the percentage of B cells and a decrease in the percentage of CD3^+^ T cells were observed, along with fewer total lymphocytes in the LNs of FTY720-treated mice in comparison to vehicle-treated mice. While FTY720 stops the egress of lymphocytes from LNs to the circulatory system, we found no evidence of increased numbers of lymphocytes in the LNs. In fact, our data indicate that there were fewer lymphocytes in LNs from FTY720-treated mice. While the relocation of these cells from the peripheral blood and LNs to the spleen seems like a reasonable explanation, we have not yet been able to confirm this.

Surprisingly, FTY720 did not prevent or reduce the primary stimulation and clonal expansion of the autoimmune T-cell response in vivo. Draining LNs from the FTY720-treated mice contained a higher percentage of CII-specific autoimmune T cells in comparison to control mice, although total numbers were similar to controls because of the smaller LNs in the treated mice. However, despite their clonal expansion in vivo, the CII-specific T cells from the FTY720 mice were unresponsive to restimulation with the CII autoantigen in vitro at time points that represent the peak of in vivo T-cell expansion [28]. While we cannot completely rule out an inhibitory effect of FTY720 directly on the autoimmune Th cells, primary stimulation and clonal expansion of the autoimmune T-cell population following immunization clearly occur in vivo in the presence of this drug. This lack of proliferative recall response was not the result of FTY720-altered antigen-presenting cell function. Antigen-presenting cells purified from FTY720-treated mice were fully capable of stimulating CII-specific T cells from untreated mice, and T cells from FTY720-treated mice remained unresponsive when stimulated with antigen-presenting cells from untreated mice. These results differ somewhat from the results reported by others who have shown that FTY720 alters the function of dendritic cells, rendering them tolerogenic for T cells [34, 35]. The antigen-presenting cells from the FTY720-treated animals in our study were derived from LN cells and were not solely dendritic cells. Collectively, this population of antigen-presenting cells functioned sufficiently to restimulate the CII-specific T cells from untreated mice, indicating no loss of function due to treatment with FTY720. Taken together, these data imply that the CII-specific T cells from the treated mice were anergic, and this concept was supported by the reversal of their unresponsive state by the addition of IL-2 to the antigen stimulation assays.

Our data and recent studies by others [20, 24, 36] indicate that FTY720 has an effect on Treg cell function. Analysis of LN cells of FTY720-treated mice revealed that the percentage of CD4^+^CD25^+^Foxp3^+^ Treg cells was twofold higher in comparison to the percentage in control animals, and this resulted in a 2.5 times higher ratio of Treg/Th cells in vivo. In addition, the function of the Treg cells from FTY720-treated mice was enhanced on a per-cell basis compared with Treg cells from control mice. That these FTY720-mediated changes in Treg cells played a major role in the regulation of the CII-specific T-cell response and the inhibition of autoimmune arthritis was demonstrated by our Treg depletion studies. Depletion of Treg cells in vivo before FTY720 treatment and disease induction prevented the induction of anergy in the CII-specific T cells and neutralized the FTY720 inhibition of autoimmune arthritis. Thus, it appears that the increased Treg ratio and enhanced regulatory function of Treg cells are a major mechanism by which FTY720 inhibits the development of autoimmune arthritis in this model. The Treg depletion studies also indicate that FTY720 does not directly turn off Th cells, as Treg-depleted FTY720-treated mice developed a significantly higher incidence of disease in comparison to the FTY720-treated nondepleted mice, and anti-CD25 treatment in the presence of FTY720 completely restored the T-cell proliferative response. These data are consistent with the observations that S1P binding to S1PR delivers a negative signal to Treg cells that inhibits not only their generation but also their regulatory activity by inducing activation of the Akt/mammalian target of rapamycin (mTOR) pathway [15, 37]. This conclusion is also supported by studies demonstrating that mice deficient in expression of S1P1R have increased numbers of nTreg cells, whereas Tg overexpression of S1P1R greatly reduces the numbers of Treg cells, and these S1P1 Tg mice develop systemic autoimmunity [37]. Finally, this relationship between S1P/S1PR binding and the downregulation of Treg activity may be a significant factor in the pathogenesis of RA. Synovial fluid from affected joints of patients with RA has high concentrations of S1P [38]. Given that the activity of Treg cells from the RA synovium is low [39], it is likely that S1P binding to Treg cells is at least one mechanism by which the Treg function in RA is downregulated, thus enabling the perpetuation of the autoimmune T-cell response in this disease.

## Conclusions

In addition to preventing the recirculation of lymphocytes from the LNs to the peripheral blood, FTY720 appears to have a significant effect on the function of lymphocytes. As a partial agonist for S1PR, FTY720 differentially alters B-cell and T-cell trafficking and both increases the Treg/Th ratio in LNs and enhances the regulatory function of the Treg cells. Similarly, there appears to be a differential effect of S1P binding to S1P1R expressed on Treg and naive T cells. Binding of S1P by conventional T cells quickly downregulates S1P1R expression, while Treg expression of S1P1R is slowly reduced [37]. S1P binding inhibits the suppressive activity of nTreg and induced Treg cells by inducing activation of the Akt/mTOR pathway [15]. The net effect appears to be that, during an immune response, Treg cell function is suppressed longer while activation of naive T cells progresses quickly. FTY720 blocks the binding of S1P, essentially reversing the homeostatic state; that is, Treg cell function is upregulated. Therapeutically, the net effect is suppression of the Th cell response, a goal in the treatment of autoimmune diseases.

In our studies, FTY720 was an effective therapeutic agent for the inhibition of autoimmune arthritis, reducing both the severity and the incidence of disease. This drug had the most pronounced effect on the recirculation of CD3^+^CD4^+^ cells, reducing their levels to <10 % in the peripheral blood. Surprisingly, FTY720 did not inhibit the in vivo generation of the clonally expanded population of CII-specific autoimmune T cells. However, at their peak expansion in vivo, these T cells were unresponsive to restimulation in vitro. Given the higher Treg/Th ratio in the LNs and the enhanced function of the Treg cells from the FTY720-treated mice, our data indicate that at least one significant mechanism by which this drug inhibits disease is through Treg cells. While FTY720 has been shown to be useful clinically for multiple sclerosis [22, 23], systemic administration of this drug has potential deleterious side effects that have to be monitored. Currently, it is considered a second line of defense drug for the treatment of multiple sclerosis, in part because of these concerns. Given our and others’ determination that this drug potentiates Treg activity, a means of site- or cell-specific delivery of this drug to Treg cells may be its most beneficial use in the treatment of autoimmunity. Such an approach would likely decrease the amount of drug required for treatment as well as improve the overall efficacy of FTY720 in the treatment of autoimmune diseases.

## Additional file

Additional file 1:
**Specificity of DR1-CII tetramer for binding to DR1-restricted, CII-specific, CD4** ^**+**^ **T cells.** T cells specific for the CII_257–274_ or HA_306–318_ peptides were incubated with the DR1-CII tetramer labeled with PE as described in the Methods section, washed, and analyzed by flow cytometry. A minimum of 10,000 cells were analyzed, and the data were gated using forward scatter vs. 90-degree light scatter. **a** CII_257–274_-specific T-cell hybridoma DR1-CII-E174 and HA_306–318_-specific T-cell hybridoma DR1-HA-8. Only the CII-specific hybridoma bound the DR1-CII tetramer. **b** CII_257–274_-specific T-cell hybridoma DR1-CII-18 and HA_306–318_-specific T-cell hybridoma DR1-HA-3. Only the CII-specific hybridoma bound the DR1-CII tetramer. (DOC 63 kb)

## Abbreviations

Ab: antibody
APC: allophycocyanin
CFA: complete Freund’s adjuvant
Cy: cyanine
DAPI: 4′,6-diamidino-2-phenylindole
EAE: experimental autoimmune encephalomyelitis
FITC: fluorescein isothiocyanate
FTY720: fingolimod
HLA: human leukocyte antigen
IgG: immunoglobulin G
IL: interleukin
LN: lymph node
mTOR: mammalian target of rapamycin
nTreg: natural regulatory T cell
PBL: peripheral blood lymphocyte
PBS: phosphate-buffered saline
PCR: polymerase chain reaction
PE: phycoerythrin
PerCP: peridinin chlorophyll
RA: rheumatoid arthritis
S1P: sphingosine-1-phosphate
S1PR: sphingosine-1-phosphate receptor
SLE: systemic lupus erythematosus
TCR: T-cell receptor
Tg: transgenic
Th: helper T cell
Treg: regulatory T cell
