# Supplementary material for: The CII-specific autoimmune T-cell response develops in the presence of FTY720 but is regulated by enhanced Treg cells that inhibit the development of autoimmune arthritis
Source: Arthritis Res Ther. 2016 Jan 12;18:8. doi: 10.1186/s13075-015-0909-6 (PMC4718028; doi:10.1186/s13075-015-0909-6)
Supplement: Additional file 1: — Specificity of DR1-CII tetramer for binding to DR1-restricted, CII-specific, CD4 + T cells. T cells specific for the CII257–274 or HA306–318 peptides were incubated with the DR1-CII tetramer labeled with PE as described in the Methods section, washed, and analyzed by flow cytometry. A minimum of 10,000 cells were analyzed, and the data were gated using forward scatter vs. 90-degree light scatter. a CII257–274-specific T-cell hybridoma DR1-CII-E174 and HA306–318-specific T-cell hybridoma DR1-HA-8. Only the CII-specific hybridoma bound the DR1-CII tetramer. b CII257–274-specific T-cell hybridoma DR1-CII-18 and HA306–318-specific T-cell hybridoma DR1-HA-3. Only the CII-specific hybridoma bound the DR1-CII tetramer. (DOC 63 kb) [file 13075_2015_909_MOESM1_ESM.doc]

**Additional file 1**. Specificity of DR1-CII tetramer for binding to DR1-restricted, CII-specific, CD4+ T cells. T cells specific for the CII(257-274) or HA(306-18) peptides were incubated with the DR1-CII tetramer labeled with PE as described in Materials and Methods, washed, and analyzed by flow cytometry. A minimum of 10,000 cells were analyzed, and the date were gated using forward vs 90° light scatter. A. CII(257-274)-specific T-cell hybridoma DR1-CII-E174 and HA(306-18)-specific T-cell hybridoma DR1-HA-8. Only the CII specific hybridoma bound the DR1-CII tetramer. B. CII(257-274)-specific T-cell hybridoma DR1-CII-18 and HA(306-18)-specific T-cell hybridoma DR1-HA-3. Only the CII specific hybridoma bound the DR1-CII tetramer.
